# Detection of Hepatitis B Virus in Used Razor Blades by PCR

**Published:** 2010-03-01

**Authors:** Cafer Eroglu, Muammer Zivalioglu, Saban Esen, Mustafa Sunbul, Hakan Leblebicioglu

**Affiliations:** 1 Department of Clinical Microbiology and Infectious Diseases, School of Medicine, Ondokuz Mayis University, Samsun 55139, Turkey

**Keywords:** Hepatitis B Virus, Transmission, PCR, Prevention

## Abstract

**Background and Aims:**

Hepatitis B virus (HBV) infection ranks among the most devastating health problems in the world.The most probable transmission routes of HBV are blood contact, sexual, and horizontal transfer. Other sources of HBV transmission are razor sharing, beauty treatments, tattooing, piercing, and manicures and other chiropody treatments.Many infections have been reported in South-East Asia, where barbers commonly share and reuse razors. Detection of HBV DNA in contaminated devices such as razor blades is important in the demonstration of transmission routes and indirect estimation of HBV prevalence in specific subpopulations such as barbershop clientele. Therefore, we aimed to detect the presence of HBV contamination on razor blades by nucleic acid testing.

**Methods:**

Used razor blades (n = 151) were purchased from different barber’s shops. Used razor blades purchased from chronic HBV patients (n = 8) were included as a positive control. The amplification and detection of HBV DNA was carried out by a semi-nested PCR method in a thermal cycler.

**Results:**

The presence of HBV DNA was found in 10 (6.6%) used razor-blade samples by the detection of a specific positive band with agarose gel electrophoresis.

**Conclusions:**

In conclusion, used razor blades may be contaminated with HBV, and the practice of sharing used razor  blades may pose a risk of transmission. Nucleic acid detection methods involving PCR can be used to detect HBV contamination of razor blades. HBV control and prevention programs should educate barbers about the importance of contagious diseases, proper sterilization techniques, and avoiding reuse and sharing of contaminated equipment and supplies such as razor blades. As an infection control measure, prohibition of razor reuse can reduce the spread of HBV infection in rural areas, where the practice is often common at barbershops.

## Introduction

Hepatitis B virus (HBV) infection ranks among the most devastating health problems in the world [1]. The prevalence of HBV is high in Southeast Asia, China, and Africa, and more than 8% of the inhabitants of these areas are chronic carriers of the virus because of either neonatal (vertical) or horizontal transmission. In contrast, North America, Western Europe, and Australia have low levels of endemicity because only a minority of people are exposed to the virus, mostly by horizontal transmission among young adults [1]. Within the different geographical regions of Turkey, however, the prevalence of infection and carriage of HBV is highly variable; for instance, whereas the carriage ratio is approximately 6% in western parts of the country, the ratio can be as high as 12.5 to 14.3% in eastern and southeastern regions [2][3][4].

Worldwide, the route of HBV transmission is mainly parenteral, although perinatal, sexual,household, and occupational transmission can occur. In Turkey, the most common transmission route is blood contact (41.1% of cases), whereas the routes for the remaining cases are unknown (49.4% of the cases) in Turkey [5]. Other possible sources ofHBV infection include sharing razor blades, beauty treatments, tattooing, piercing, and manicure and other

Despite the fact that needles and razor blades can transmit HBV, a feasible detection method (e.g.inoculation of cell cultures or animals) for HBV does not exist to date [6][7]. Although several researchers have tried to start different cell-culture systems such as HepG2, others could not reproduce the results [8][9][10]. In a literature search in PubMed, we did not find any specific molecular study designed to detect HBV DNA on razor blades. Detection of HBV DNA in contaminated devices such as razor blades is important in the demonstration of transmission routes and indirect estimation of HBV prevalence in specific subpopulations such as barbershop clientele. Therefore, we aimed to detect the presence of HBV contamination on razor blades by polymerase chain reaction (PCR) based nucleic acid testing.

## Material and Methods

### Razor-blade samples 

Used razor blades (n = 151) were purchased from different barbershops in Samsun, Turkey. Used razor blades purchased from chronic HBV patients (n = 8) were used as a positive control. Identifying information for each blade was recorded, and each blade was put in a single DNAse-free and RNAsefree tube (15 mL). Each used-razor-blade sample was stored separately at -20°C until the blades were studied. We prospectively collected each subject’s name, surname, sex, and ages.

### DNA isolation

In the first step of DNA isolation, 200 μL of distilled water, 40 μL of proteinase K, and 200 μL of binding buffer were added to each tube, in turn. Tubes were vortex mixed vigorously. After that, HBV DNA was isolated with a high pure PCR template preparation kit (Roche Diagnostics Mannheim, Germany) according to the instructions of the manufacturer. To elute the HBV DNA, a 50μL elution buffer was used.

### PCR

**Figure 1:**
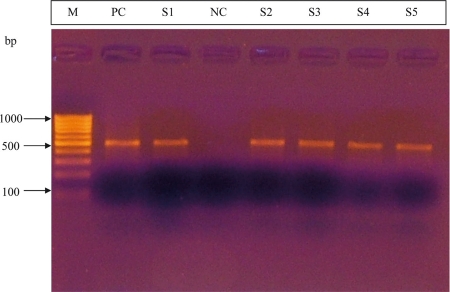
Agarose gel image of the second round PCR products. Lane 1: marker (M) (100 bp), lane 2: positive control (PC), lane 4: negative control (NC) and lanes 3, 5-8: positive samples (S).

The amplification and detection of HBV DNA was carried out by a semi-nested PCR method in a thermal cycler. For amplification, 5 μL of HBV DNA were added to a 45-μL reaction mixture. HBV primers (P8: 5’-CTCCAGTTCAGGAACAGTAAACCC -3’, primer 840: 5’-ACCCCATCTTTTTGTTTTGTTAGG -3’)were used for the first round of PCR. The primers 377 (5’- GGATGTGTCTGCGGCGTTT -3’) and 840 were used in the second round of PCR (11, 12). The PCR reaction mixture contained 200 mM of each nucleotide triphosphate, 0.5 μM of each primer, 1.5 mM of MgCl2, and 2 units of Hot Start Taq DNA polymerase (MBI Fermentas) in a 50-μL total reaction volume. Samples were thermocycled (GeneAmp PCR System 9700, Applied Biosystems) for 35 cycles (1 min at 95°C, 1 min at 55°C, 2 min at 72°C). Then, 2 μL of the PCR products were added to the second round PCR mixture. Second-round PCR products were separated in a 1.5% agarose gel, then stained with ethidium bromide and viewed under UV light. A result was considered positive when a band of the appropriate size was visible in the gel (see Fig. 1). Standard procedures for reducing contamination were strictly followed.

## Results

All 151 subjects were male, and the mean age ± standard deviation (SD) was 34.6 ± 10.0 years. HBV DNA was detected in 10 (6.6%) razor-blade samples by the detection of a positive band with the agarose gel electrophoresis (Fig. 1). All used razor blades (n = 8) purchased from chronic HBV patients were also positive.

## Discussion

The whole virion, or Dane particle, is a 42-nm sphere that contains a core, or nucleocapsid, enclosing the DNA [13]. Other HBV particles are small spheres and rods with an average width of 22 nm and contain a great excess of envelope material. Only the Dane particle is infectious and carries DNA. Human blood plasma is the main virus reservoir, and sera from certain HBV carriers-even in dilutions of 1:100,000,000-are still able to infect chimpanzees when administered intravenously. Studies using chimpanzees indicate that, as few as 1-10 virions are infectious when inoculated intravenously [14]. These studies suggest that PCR positivity in razor-blade samples could contain sufficient inoculum or Dane particles to transmit the HBV infection.

We considered that used razor blades could have been contaminated with HBV through the normal bodily fluids of infected patients. Unfortunately, as of this writing, there are no feasible infectivity assays available to validate the route of HBV infection, either in cell cultures or in small animals, although several authors have reported different cell culture systems such as HepG2 [8][9][10]. However, others could not reproduce the results of these studies [10]. Thus, there is a need for a sensitive and specific method to detect the presence of HBV on razor blades.

In this study, HBV DNA was detected in 10 (6.6%) used razor-blade samples. This positivity rate is plausible because Turkey is a country with an intermediate endemicity for HBV. The rate indicates that sharing razor blades can be risky in endemic areas. Particularly in hyperendemic areas, sharing razor blades should be considered a very important risk factor because of the higher numbers of HBV carriers. Our method can be used for the detection of HBV presence in razor blades and other similar devices. With this method, the extent of risk can be shown in detail, and appropriate infection control measures can be implemented.

The main transmission routes of HBV are parenteral and perinatal. Sexual, household, and occupational transmissions are also important [5]. HBV infection sources such as razor sharing, beauty treatments, tattooing, piercing, and manicure/chiropody may also be important risk factors in developed and undeveloped areas [7][15][16][17][18]. HBV DNA can be found in different bodily fluids of HBV infected patients. In particular, reuse or sharing of contaminated materials like razor blades can be considered important routes for the transmission of HBV infection. Razor sharing or reuse is a statistically significant, independent risk factor for transmission of hepatitis in developed and undeveloped countries [7][15][17][18]. It has been reported that hepatitis B viruses can live for several days in dried blood on table surfaces, needles, syringes and razors [19].

Despite this knowledge, these two practices continue to be common many parts of the world.  For instance, a survey of barbers in India and Pakistan [16][20] revealed that many used razor sharing or reuse. Although 64% of barbers claimed use of new blades for each customer, only 19% declared sterilization of their instruments. Janjua et al. found that 46% of barbers in Pakistan reused blades without sterilization [16]. Although the frequency of exposure was low in general, for individuals who received daily shaves from barbers, the frequency of exposure was very high. They concluded that the probability of transmission increases with the frequency of reuse [16].

The unawareness among barbers and healthy people at risk of contracting HBV through sharing razor blades may weaken the control of hepatitis B transmission, and end up with an increase in prevalence even in low-endemic areas. Candan et al.  found that barbers in Turkey may often be exposed accidentally to the blood and bodily fluids of their customers; indeed, the authors found that the prevalence rates of HBV and hepatitis C virus were higher in barbers (39.8 and 2.8%, respectively) than in a comparison group (28.3 and 1.1%, respectively) [15]. HBV control and prevention programs focusing on the importance of contagious diseases, proper sterilization techniques, and avoiding reuse and sharing of contaminated equipment and supplies such as razor blades should also target barbers. General reduction of these exposures and assuring sterile practices are logical goals for intervention [19]. It is essential and urgent to promote awareness of these risks among everyone, especially barbers, men who frequent barber shops, and the public authorities, and to ban barbers from the illegal practice of taking medicine for their own protection. In addition, razor reuse should be forbidden at barbershops.

In the literature, there are few articles about hepatitis transmission and beauty treatment [18][21][22]. It has been shown that certain beauty treatments play an important role in the spread of HBV and HCV. Mariano et al. predicted that the proportion of cases of parenterally transmitted viral hepatitis due to such exposure will increase in the coming years because beauty treatments such as tattooing and body piercing are increasing in popularity.  They underline the need for increased use of disposable materials and effective sterilization of instruments during beauty treatments. Implementation of training and compulsory, routine controls for professional and nonprofessional personnel involved in these procedures may lead to a significant reduction of parenterally transmitted acute viral hepatitis cases [18].

Because all used razor blades collected from chronic HBV patients were positive in our study, special efforts should be made to educate patients with chronic hepatitis B infection about the risk of spreading HBV via razor blades. They should particularly be warned of the risks of sharing personal articles like razor blades.

In conclusion, nucleic acid detection methods can be used for the detection of HBV in devices such as razor blades. Reuse of razor blades is an important route for transmission of HBV infection, and general reduction of these exposures and assuring sterile practices are logical goals for intervention and prevention of hepatitis B infection. Future studies should focus on how to detect biologically active viruses quantitatively in risky devices.
